# Are Endovascular Interventions for Central Vein Obstructions due to Cardiac Implanted Electronic Devices Effective?

**DOI:** 10.3389/fsurg.2018.00049

**Published:** 2018-07-30

**Authors:** Charalampos Sotiriadis, Stephanie Volpi, Pauline Douek, Amine Chouiter, Olivier Muller, Salah D. Qanadli

**Affiliations:** Cardiothoracic and Vascular Unit, Department of Radiology, University Hospital of Lausanne, Université de Lausanne, Lausanne, Switzerland

**Keywords:** CIED, lead exchange, central venous occlusion, angioplasty, stenting

## Abstract

**Objective:** One of the late-onset complications of cardiac implanted electronic devices (CIEDs) is central venous obstruction (CVO). The aim of this study was to investigate the feasibility, efficacy, and safety of endovascular treatment of CIED-related CVOs.

**Methods:**Eighteen patients who underwent endovascular management of their device-related CVO were reviewed. Patients were classified into three groups: Group I patients were asymptomatic and needed lead replacement; Group II patients presented with symptomatic CVO without lead dysfunction, and Group III patients were referred with both symptomatic CVO and lead dysfunction. A treatment strategy involved recanalization and balloon angioplasty for Group I and angioplasty/stents for Groups II and III. Technical success, clinical success, complications, and long-term follow-up were assessed.

**Results:** Thirteen patients were in Group I, four in Group II, and one in Group III. Technical and clinical success was achieved in 17 patients (94%). No major complications were reported. Restenosis was observed in two patients at 40 and 42 weeks of follow-up, and these patients were successfully treated with angioplasty.

**Conclusion:** Endovascular management of CVO due to CIED is a safe and efficient technique. Plain balloon angioplasty is sufficient for lead replacement purposes, while stenting is needed for symptomatic CVO to achieve good long-term patency.

## Introduction

Since their introduction in 1960s, transvenous cardiac implanted electronic devices (CIED) (which include pacemakers, cardioverter-defibrillators, and cardiac resynchronization therapies) continue to provide obvious benefits for the management cardiac rhythm disorders and heart failure (1). They are, however, associated with such complications as device dislodgement, perforation, infection, migration, and vein stenosis or thrombosis (2). With the growing number of CIED implantations, complication prevention, and management are a crucial clinical issue. Central venous obstruction (CVO) is the most important complication. The reported incidence of CVO after CIED implantation varies widely (3).

CVOs are usually asymptomatic (4). It was reported that only 50% of patients with CVOs are symptomatic (5). The most severe clinical consequence is the development of superior vena cava syndrome (SVCS) (5). Fortunately, the incidence of CIED-induced SVCS is rare (less than 0.1%) (6).

There are various approaches for managing CIED-induced CVOs, which can be applied as monotherapies or as combotherapies. Pharmacological or pharmacomechanical management is preferred in cases of recent thrombosis (7). Endovascular or surgical treatments are indicated when a CVO is diagnosed (8).

The technique of endovascular management of benign causes of SVC syndrome has been described in a previous report (9). An important issue within this patient population is whether CIED leads have to be removed before stent placement. Several studies have demonstrated no early complications related to the metal-to-metal contact between the lead and stents *in vivo* (10). However, there is still a concern about the long-term effects (11).

Furthermore, the patency of access veins is another critical issue. This is because the incidence of CVOs in patients who are candidates for CIED is as high as 11% prior to the first device (12). It is also because many patients after CIED implantation will undergo additional procedures for lead revisions or upgrades. Currently, future clinical challenges relate to identifying patients who will benefit from the detection of silent CVOs before CIED implantation, identifying patients that should have superior vena cava system assessment before lead revision, and determining how to manage chronic CVO in symptomatic and asymptomatic patients with CIED.

The aim of this single-center study was to evaluate the efficacy and safety of percutaneous endovascular management of CIED-related CVOs.

## Materials and methods

### Patient selection

We reviewed all patients with history of a CIED, who were referred to the Radiology department for CVO from February 2005 to April 2016. Patients with hemodialysis arterio-venous shunts were excluded. Our institutional ethics committee approved this study. Written consent was obtained from all patients for each procedure. From this population of patients, we found 18 patients (13 men and 5 women) who had been treated with percutaneous endovascular techniques. The mean age was 69 ± 10 years (range: 48–85 years). Sixteen patients had a pacemaker, and only two had a defibrillator. Table 1, demonstrates demographic and clinical information of the patients. They have been classified into three groups based on the presence of symptoms and/or CIED dysfunction as follows:

1. Group I: Patients were asymptomatic, but it was suspected that they had CVOs due to the failure of pacemaker lead extraction and/or upgrade or because of a failure to insert a peripheral central line.
2. Group II: Patients presented with CVO-related symptoms. No dysfunction of the CIED existed.
3. Group III: Patients were symptomatic, and CIED had to be changed due to dysfunction.

**Table 1:** Patient's demographic and clinical characteristics.

**Patient number/age**	**Sex**	**CIED type**	**Indication for CIED placement**	**Side**	**Type**	**Vein involved**	**Symptoms**	**Comorbidity**
1/52 years	F	Df	Ventricular fibrillation	L	II	In, SVC	Headache, dyspnea, pectoral telangiectasia	Systemic lupus erythematosus
2/64 years	M	Pc	Sick sinus syndrome	L	II	In-SVC junction	Left arm edema	No
3/69 years	M	Pc	Sick sinus syndrome	L	IV	SVC	Head and neck edema	Hypertension
4/72 years	M	Pc	Atrioventricular block	L	II	SVC	Upper body edema	COPD
5/76 years	M	Pc	Sick sinus syndrome	L	IV	SVC	Upper body edema	No
6/68 years	M	Pc	Dilated cardiomyopathy with heart failure	R	IV	Sc	No	Renal insufficiency
7/48 years	M	Pc	Sick sinus syndrome	R	II	Sc, In	No	Cirrhosis
8/67 years	M	Pc	Bradycardia	L	IV	Sc, In	No	Hypertension
9/82 years	F	Pc	Syncope	L	II	In-SVC junction	No	Obesity
10/62 years	F	Pc	Sick sinus syndrome	L	II	Sc	No	PAD
11/66 years	M	Pc	Ventricular tachycardia	L	IV	In-SVC junction	No	No
12/85 years	M	Pc	Sick sinus syndrome	L	II	Sc, In	No	PAD
13/64 years	M	Pc	Atrioventricular block	L	IV	Sc, In	No	COPD
14/83 years	M	Df	Ventricular tachycardia	L	II	Sc, In	No	PAD
15/70 years	M	Pc	Bradycardia	L	IV	Sc, In	No	Renal insufficiency
16/75 years	M	Pc	Sick sinus syndrome	R	IV	Sc, In	No	No
17/65 years	M	Pc	Syncope	L	II	Sc, In	No	Cirrhosis
18/78 years	M	Pc	Sick sinus syndrome	L	IV	In-SVC junction	No	No

*Df, Defibrillator; Pc, Pacemaker; In, Innominate vein; Sc, Subclavian vein; SVC, Superior Vena Cava; COPD, chronic obstructive pulmonary disease; PAD, peripheral arterial disease*.

All patients were evaluated with conventional venography. In symptomatic patients, direct CT venography (13) was also performed in order to evaluate collateral supplies and define jugular drainage. SVCs and CVOs were classified based on the location and degree of obstruction as follow:

- Type I: There is an isolated stenosis of the SVC;
- Type II: Corresponds to a central vein stenosis with or without extension to the SVC;
- Type III: A chronic total occlusion of the SVC;
- Type IV: Corresponds to a chronic total occlusion of central veins with or without extension to the SVC.

### Technique description

**Figure 1:**
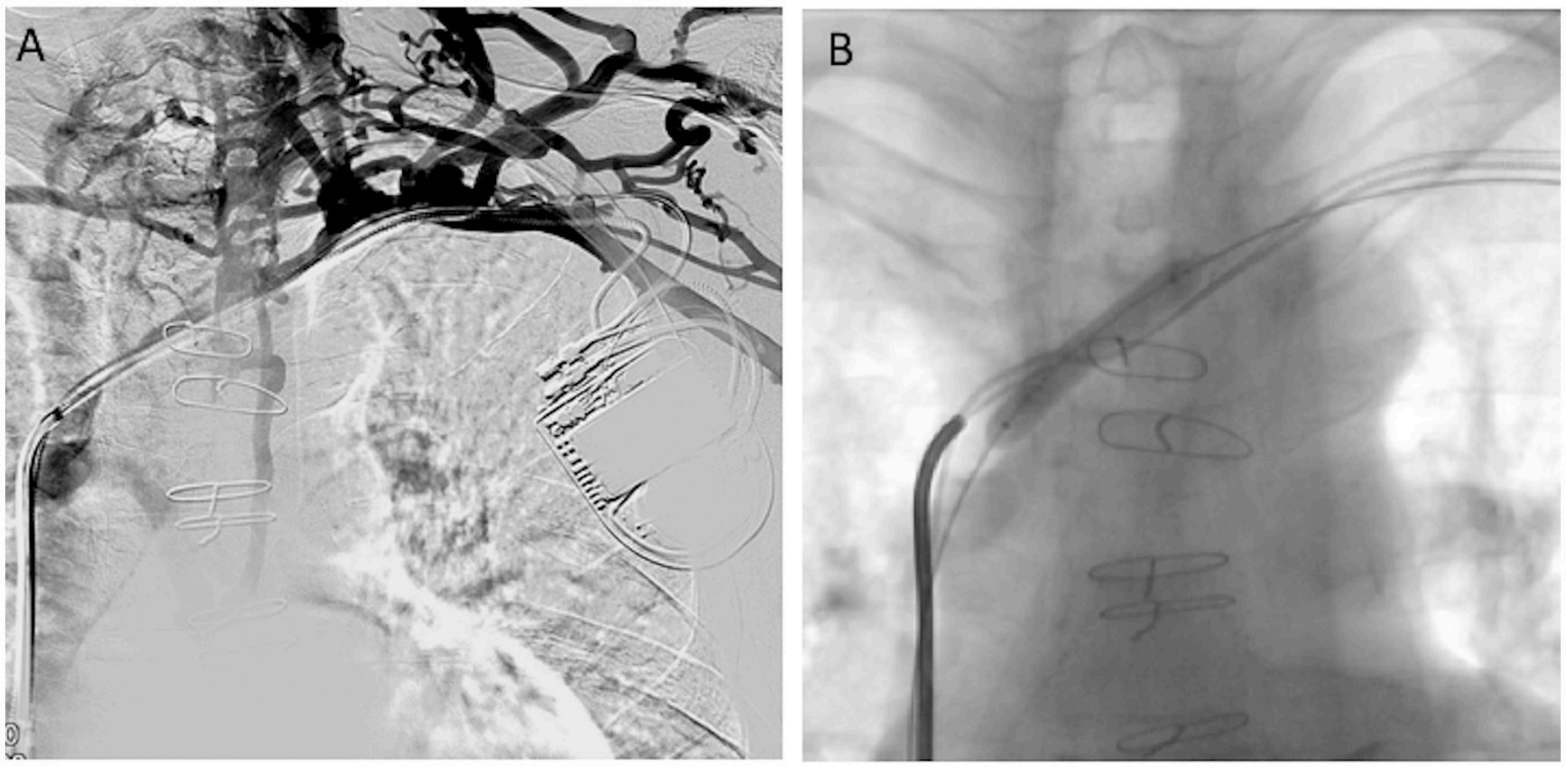
A 70-year-old asymptomatic man (Group I) living with a pacemaker for 3 years was candidate for lead replacement due to dysfunction. Venography was considered because of failure to advance new leads in the Cath-Lab. **(A)** Venography revealed occlusion of the left innominate vein with the presence of collateral veins. **(B)** Left innominate vein angioplasty was performed in order to facilitate lead passage.

Two radiologists with 20 and 25 years of experience in vascular interventions treated the patients. Before any procedure venography was performed, the ipsilateral basilic or brachial vein was punctured under ultrasound guidance, and a 4-French 10-cm introducer sheath was inserted. Based on venography results, a single or combined venous approach (brachial and femoral) was used. In asymptomatic patients, hydrophilic guidewire-based (Terumo European N.V., Leuven, Belgium) recanalization was performed through the same access (brachial) or through femoral access in four patients (30%) based on the radiologist's preference. A 6-French introducer sheath was used. After the administration of 5,000 IU of heparin, angioplasty was performed starting with a small-sized balloon, and this was gradually upgraded in size. The goal was to dilate the vein at this level to facilitate passage of new leads or catheters (Figure 1). Typically, ambulatory patients remained for 4 hours in the radiology department for monitoring before discharge. Balloon angioplasty with progressive dilatation was performed using 4 to 10 mm high-pressure balloons.

**Figure 2:**
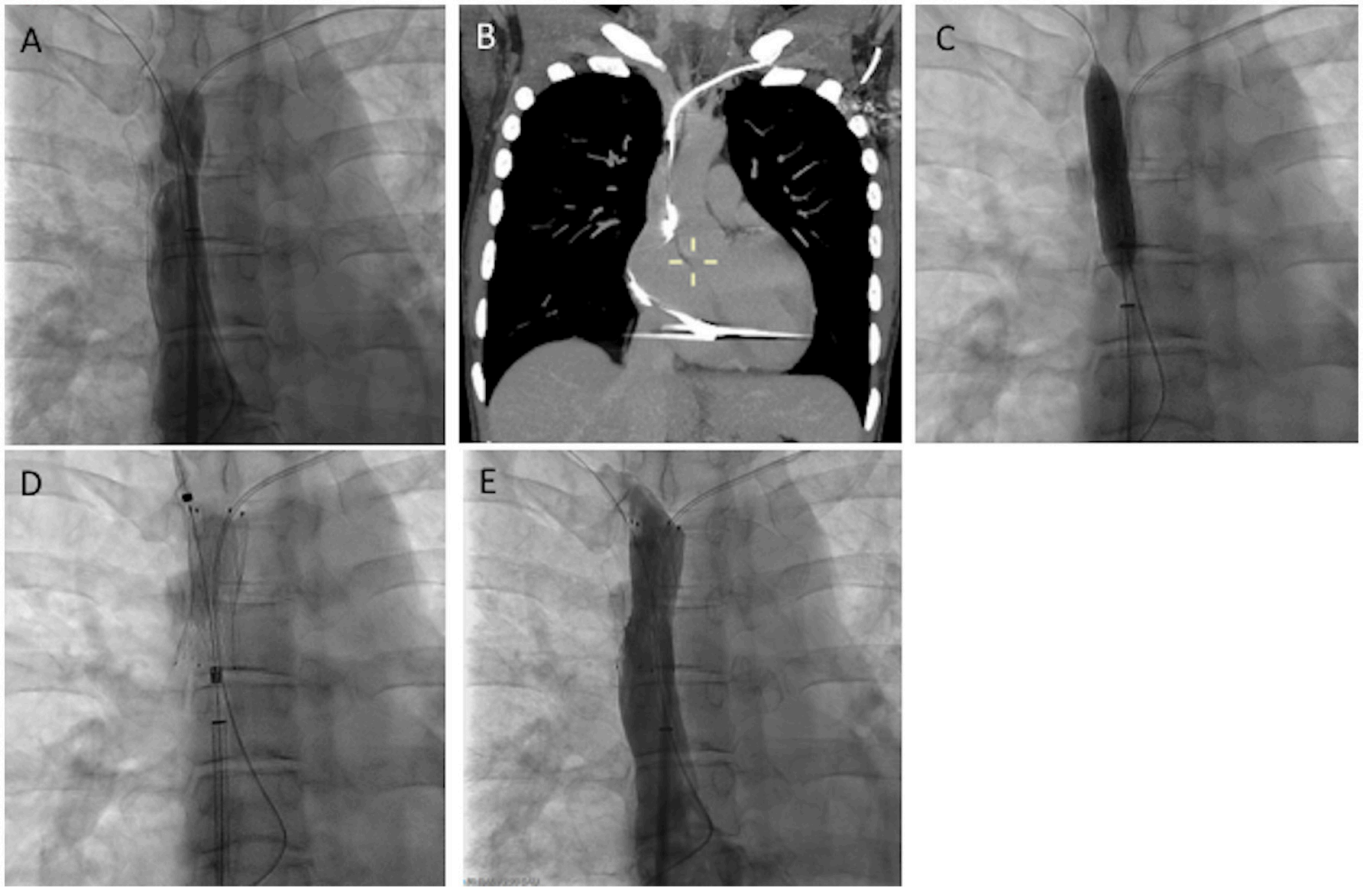
52-year-old woman with a defibrillator for the past 5 years presented with headache and dyspnea for 20 months. No dysfunction of the CIED was noted (Group II). **(A)** Cavography of the SVC was performed, revealing severe stenosis of the SVC. **(B)** CT images of MIP coronal reconstruction, showing fibrotic stenosis of the left innominate vein. **(C,D)** Progressive balloon angioplasty and stenting of the SVC was performed without lead removal. **(E)** Cavography showed normal flow through the stent. The patient's symptoms disappeared completely 3 days later.

In patients with SVC syndrome, the common femoral vein was punctured, and a 10-French 65-cm introducer sheath was inserted. Venography was performed via brachial access through a 4-French introducer sheath or through the 5-French vertebral curve catheter introduced via femoral access. Recanalization, pressure measurements, and gradual angioplasty were performed as described by Qanadli et al. (9) and Breault et al. (14) (Figure 2). Progressive angioplasty was performed using 6 to 14 mm high-pressure balloons. In all patients, SinusXL stents (OptiMed, Ettlingen, Germany), which varied from 16 to 24 mm, were used because of recoil and residual stenosis (Table 2). In order to avoid proximal displacement, the stent was under-dilated. Patients were monitored post-operatively for at least 24 h in order to manage any eventual heart failure. Oral anticoagulation treatment was administered for a 6-month period (Syntrom).

**Table 2:** Details of material and complications in symptomatic patients.

**Patient number/age**	**Sex**	**Ballon size in mm**	**Sinus XL stent size in mm**	**Intrastent symptomatic restenosis**	**Complication**
1/52 years	F	6, 8, 10	20	In 40 weeks	No
2/64 years	M	10, 12	22	No	No
3/69 years	M	8, 10	16	In 42 weeks	No
4/72 years	M	12, 14	24	No	No
5/76 years	M	8, 10	20	No	Early battery discharge

Technical success was defined as follows: Group I, successful recanalization of the obstruction followed by angioplasty; Group II, reestablishment of venous blood flow to a normal physiological pattern; Group III, reestablishment of venous blood flow to a normal physiological pattern and successful replacement of the lead. Clinical success was defined as follows: Group II and III, regression or resolution of symptoms 48 h to 1 week after treatment. All symptomatic patients were followed up clinically with CT venography and a systematic visit at 3 months as a standard care workflow. A telephone contact was maintained for a long-term follow evaluation for all groups of patients.

## Results

We identified 13 patients in Group I, four in group II, and one in group III. From Group II, one patient presented with swelling of the face and shortness of breath, one with swelling of the left arm, one with swelling of the face and neck accompanied by hoarseness, and one with swelling of the upper body. The patient in Group III presented with swelling of the upper body. Nine patients had Type II obstruction, and nine had Type IV obstruction. For symptomatic patients, three had Type II obstruction and two Type IV (Table 1). Asymptomatic patients were managed only by balloon angioplasty, while symptomatic patients received angioplasty and stent placement. In one asymptomatic patient with Type II obstruction, recanalization failed.

In asymptomatic patients, hydrophilic guidewire-based recanalization was performed through the same access (brachial) in seven patients (54%) and through the femoral access in five patients (38%). In one patient, both femoral and brachial access were needed. The subclavian vein was involved in 10 patients, the innominate vein in 10 patients, and the innominate vein-SVC junction in three patients.

Symptomatic patients were treated using the femoral venous approach. A brachial access was used to perform initial venography and a subsequent control series. The innominate vein-SVC junction was involved in one patient, and the SVC was involved in four patients. High pressure and short balloons were preferred over long balloons to provide spot or multiple angioplasties (if required). The deflation time was usually very long for large-diameter high-pressure balloons, which is often less tolerated by patients. We also believe that the theoretical risk of rupture might be increased with long balloons aiming to dilate the innominate vein and the SVC simultaneously. A sinus XL stent was used in all patients with a nominal diameter. In-stent remodeling with balloons was systematic. Patients in Group III were treated initially with balloon angioplasty and stenting over the ancient leads. The leads were not removed, and the new leads were inserted through the stent to the cardiac cavities.

Stenosis at the entry site with proximal extension was more often present in patients (56%). Obstructions located only at the insertion site were found in two patients (11%), and obstructions located distal to the insertion point were present in six patients (33%). Symptoms were present when stenosis involved the SVC or the innominate vein-SVC junction.

Technical and clinical success was achieved in 17 of 18 patients (94%).

No early complications were observed. In one symptomatic patient treated with a stent, the battery had to be replaced 2 years sooner than expected.

At 3 months of follow-up, all patients were asymptomatic, and in Groups II and III CTs showed stent patency without early stenosis. In one patient from Group I, the electrodes of the pacemaker had to be changed 4 years later, and cardiologists had difficulty advancing new leads through the central veins. Venography revealed subclavian vein restenosis, which was successfully treated with angioplasty.

**Figure 3:**
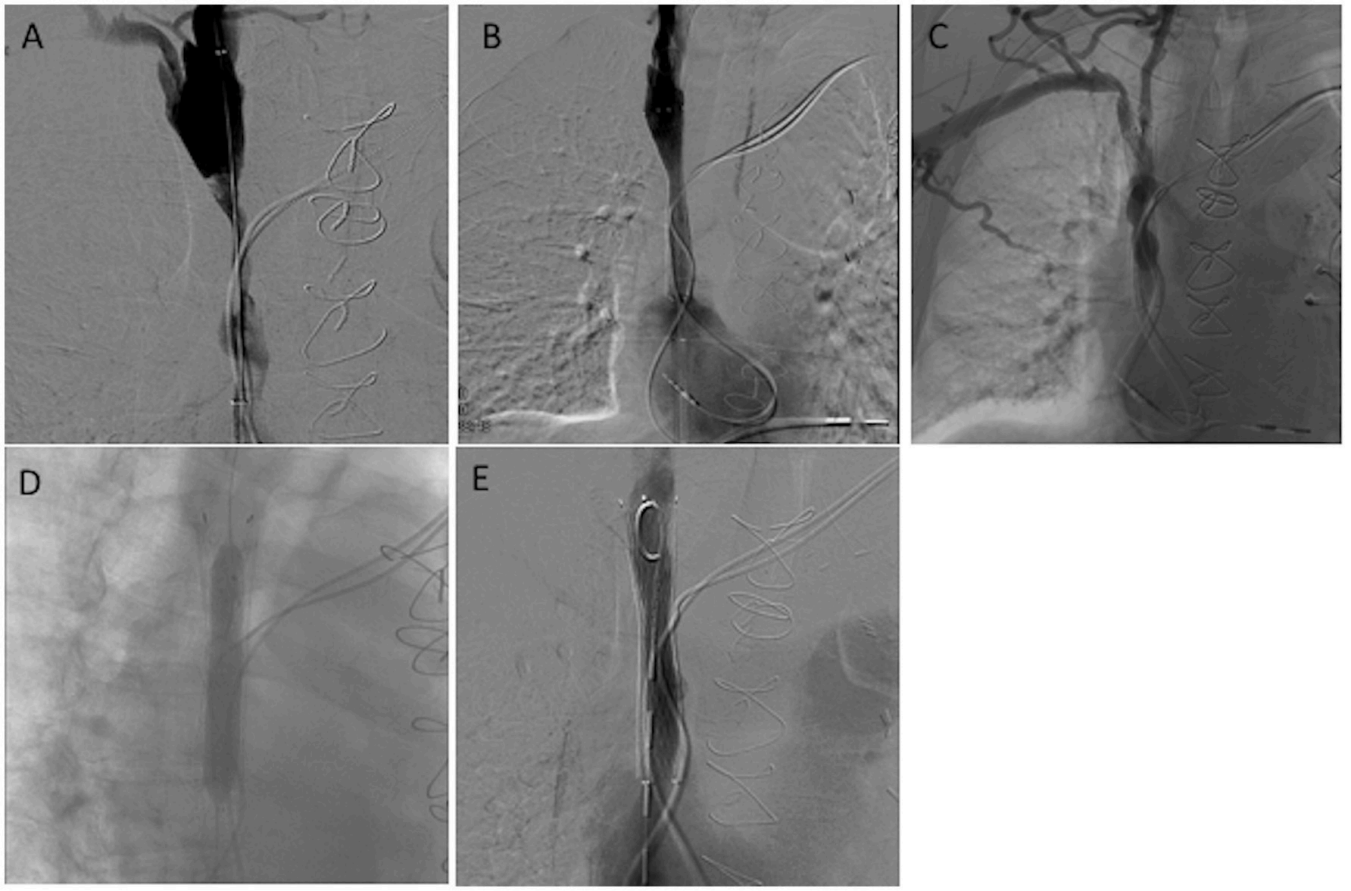
A 75-year-old man living with a pacemaker for the past 4 years complained of headache and facial edema for the last 3 months. No dysfunction of his pacemaker was found (Group II). **(A,B)** Initial venography showed severe stenosis of the SVC, which was successfully treated by angioplasty and stenting. After 40 months, the patient presented again with the same symptoms. (**C–E**) Radiological evaluation demonstrated stenosis in the SVC stent, which was successfully treated by angioplasty.

Two of the symptomatic patients presented with symptom recurrence at 40 and 42 weeks of follow-up, and they were retreated successfully with angioplasty (Figure 3). All patients have experienced no further symptoms as of today (37 ± 27 months of follow-up), which has been verified by telephone communication.

## Discussion

Our data showed that 70% of patients were asymptomatic, while about half of all CVOs were total occlusions. Symptoms seemed to be more related to obstruction extension than to obstruction degree. In the literature, CVO was observed in 14–64% of patients after pacemaker insertion (15). Total occlusions were reported in up to 26% of patients (16). More than 75% of CVOs were observed after 6 months (17).

The mechanism underpinning the development of a CVO after lead placement has been debated and is not fully understood. Endothelial disruption due to mechanical stress or infection of leads is the main factor thought to predispose a patient to developing a CVO (18). Induced inflammatory changes in the vessel wall with subsequent scarring and fibrosis might lead to progressive vessel wall and lumen remodeling resulting in vessel lumen reduction (19–22). Furthermore, thrombus formation might also play a significant role. Lonyai et al. presented a novel application of computational methods to study blood flow changes induced by pacemaker leads (23). Their results showed that lead placement could result in pockets of low blood flow velocities between leads and vessel walls, predisposing patients to vein thrombosis. The reported risk factors of CIED-related CVO include a history of device upgrade, the presence of temporary endocardial pacemaker wires before permanent implantation, lead infection, the use of dual-coil leads, hormone therapy, and a history of venous thrombosis (24). The number of leads may also contribute to this condition (19, 20). Narrowing of the vessel due to the presence of more leads and endothelial damage due to physical manipulation may make the vein susceptible to thrombosis and fibrotic scarring (25). Female gender (26) and diabetes (27) have a protective effect against the development of CVO.

The fibrotic changes in the vein wall might impact endovascular management because these changes are often responsible for the elastic recoil observed after angioplasty. In order to maintain vessel recoil, gradual balloon angioplasty under high pressure is required. The recoil is sometimes so important that even after appropriate angioplasty, the insertion of new wires is not feasible. In that case, when angioplasty has been performed, a 6-French long introducer (25 cm) is used with its tip placed distal to the obstruction. Afterwards, leads can be advanced in the cardiac cavities through this introducer. When leads are placed in the suitable position, then the introducer is removed.

Angioplasty or stenting was well-tolerated by our patients. No heart failure was noted after treatment in symptomatic patients. All patients experienced improvements in symptoms immediately after endovascular treatment. Undoubtedly, endovascular treatment may potentially be associated with lead damage. Progressive angioplasty is critical in order to avoid this potential complication. In cases of endovascular stent placement over pacemaker wires, one possible concern is the long-term effect on pacemaker function resulting from metal-to-metal contact (10, 28, 29). Isolation between the wires and the stent does exist when pacemaker leads are covered by polyurethane and by a layer of neointimal endothelialization along segments fully opposed to the vein wall. However, at points where there is no contact with the vein wall or after stress post-angioplasty, isolation may fail. Thus, a potential risk for dysfunction exists. In our five patients treated with stenting of the SVC without lead removal, one presented with early battery dysfunction. One patient was treated 7 years before, and no dysfunction of his pacemaker has been noted over this relatively long time period. These findings are consistent with those from the retrospective review of Riley et al. for the management of retained leads, which reported no adverse effects after stenting (8).

In our study, we did not try to extract wires before endovascular angioplasty in symptomatic patients. One option is lead extraction assisted with a laser method before angioplasty and stenting in order to avoid recurrence. However, this technique has important risks (22, 30, 31) and higher costs. Moreover, lead retention was not associated with symptom recurrence (8).

In symptomatic patients, venous bypass followed by surgical thrombectomy is a theoretical alternative treatment, albeit a highly invasive operation with a higher recurrence rate than percutaneous methods (8). Other alternatives include contralateral implantation of a completely new system, recanalization using laser tools, gaining access medially to the occlusion, femoral/iliac access with a femoral/abdominal pocket, and epicardial lead placement (32, 33). We do not recommend contralateral insertion, particularly in asymptomatic patients. This is because the risk of developing symptoms is high. A promising emerging method to treat cardiac rhythm disorders could be the use of leadless devices that have the advantage of less risk of CVO (34, 35).

The present study has two primary limitations: (1) the small number of patients and (2) the retrospective collection of data. Moreover, all symptomatic patients were treated using a Sinus XL stent. As a result, we have no experience with metal-to-metal interactions between pacemaker leads and other types of venous stents.

In conclusion, endovascular management of induced CVOs is a safe and efficient technique. Plain balloon angioplasty is sufficient for lead replacement purposes, while stenting is feasible and safe for symptomatic CVOs, resulting in good clinical outcomes.

## Ethics statement

This study was carried out in accordance with the recommendations of ‘Commission cantonale d'éthique de la recherche sur l'être humain, CER-VD’ with written informed consent from all subjects. All subjects gave written informed consent in accordance with the Declaration of Helsinki. The protocol was approved by the ‘Commission cantonale d'éthique de la recherche sur l'être humain, CER-VD’.

## Author contributions

CS, SQ: concept formulation, patient management, and manuscript writing/editing. SV, AC, PD: patient selection, patient management, and manuscript writing/editing. OM: manuscript writing/editing and figures.

### Conflict of interest statement

The authors declare that the research was conducted in the absence of any commercial or financial relationships that could be construed as a potential conflict of interest.
